# Cytokine, Chemokine, and Neurofilament Light Chain Signatures in LGI1 Autoimmune Encephalitis

**DOI:** 10.1002/acn3.70158

**Published:** 2025-08-08

**Authors:** Albert Aboseif, Georgios Mangioris, Binxia Yang, Vanessa K. Pazdernik, Jeffrey W. Britton, Divyanshu Dubey, Eoin P. Flanagan, Sarosh R. Irani, Gregory S. Day, Charles L. Howe, A. Sebastian López‐Chiriboga, Andrew McKeon, John R. Mills, Yahel Segal, Michel Toledano, Ivana Vodopivec, Sean J. Pittock, Anastasia Zekeridou

**Affiliations:** ^1^ Department of Neurology Mayo Clinic College of Medicine Rochester Minnesota USA; ^2^ Center for Multiple Sclerosis & Autoimmune Neurology Mayo Clinic College of Medicine Rochester Minnesota USA; ^3^ Department of Laboratory Medicine and Pathology Mayo Clinic College of Medicine Rochester Minnesota USA; ^4^ Department of Quantitative Health Sciences Mayo Clinic Rochester Minnesota USA; ^5^ Department of Neurology Mayo Clinic Jacksonville Florida USA; ^6^ Nuffield Department of Clinical Neurosciences, Oxford Autoimmune Neurology Group University of Oxford Oxford UK; ^7^ Roche Product Development‐Neuroscience, F. Hoffmann‐La Roche Basel Switzerland

**Keywords:** autoimmune seizures, biomarkers, epilepsy, faciobrachial dystonic seizures, Leucine‐rich glioma‐inactivated‐1

## Abstract

**Objectives:**

To investigate the value of cytokine, chemokine, and neurofilament light chain (NfL) concentrations in predicting relapse risk, chronic epilepsy, and functional impairment in LGI1 autoimmune encephalitis (AE).

**Methods:**

Cytokines/chemokines (IL‐1‐beta, IL‐2, IL‐4, IL‐5, IL‐6, IL‐8/CXCL8, IL‐10, IL‐12p70, IL‐13, IL‐17A, GM‐CSF, TNF‐alpha, IFN‐gamma, CXCL9, CXCL10, CXCL13, BAFF) and NfL concentrations were measured in CSF and paired serum from LGI1‐AE patients evaluated at Mayo Clinic (01/2015–02/2024), using a multiplex immunoassay system (ELLA, Bio‐Techne) and correlated with clinical outcomes. A laboratory‐based cohort of LGI1‐IgG‐positive patients and control cohorts, including patients with mixed non‐inflammatory disorders (MNID), Alzheimer's disease (AD), and temporal lobe epilepsy (TLE) were analyzed.

**Results:**

Forty‐four patients with LGI1‐AE were included; 29 (66%) were male, with a median age of 68.5 years (range, 8–85). Median time from symptom onset to CSF sampling was eight months (IQR, 3–17); 19/42 (45%) experienced a clinical relapse and 27% developed chronic epilepsy. Serum IL‐6, serum and CSF IL‐8/CXCL8, and IL‐17A were higher in LGI1‐IgG positive patients than MNID (*p* < 0.05). TLE cytokine/chemokine profiles were similar to LGI1 AE; AD patients had lower serum IL‐6 and CSF IL‐8/CXCL8 (*p* = 0.04; *p* = 0.01), and higher serum IL‐17A and GM‐CSF (*p* = 0.004; *p* = 0.01) than LGI1‐AE. Higher CSF IL‐6 and IL‐8/CXCL8 in LGI1‐AE associated with clinical relapse (*p* < 0.05) and higher CSF NfL associated with chronic epilepsy (*p* = 0.01).

**Conclusion:**

Elevations in IL‐6, IL‐8/CXCL8, and IL‐17A were identified in this LGI1‐AE cohort. CSF IL‐6, IL‐8/CXCL8, and NfL levels are potential prognostic biomarkers for risk of relapse and chronic epilepsy in LGI1‐AE.

## 1 Introduction

Leucine‐rich glioma‐inactivated‐1 (LGI1) antibodies are among the most frequently detected cell‐surface anti‐neuronal antibodies causing autoimmune encephalitis (AE) [1, 2, 3, 4]. Clinical features such as disease severity, treatment responsiveness, long‐term morbidity, and risk of relapse are variable among patients, suggesting immunobiological heterogeneity [5, 6, 7, 8]. Furthermore, despite improvement with immunotherapies, patients with LGI1‐AE may experience persistent seizures and residual neuropsychiatric features [5, 6, 7, 8, 9]. Deciphering the underlying immunology of the disease may provide insight into this heterogeneity, leading to improved prognostication and novel therapeutic strategies.

Cytokine and chemokine levels can inform diagnostic, prognostic, and therapeutic decisions in several immunologic diseases, including forms of AE [10, 11, 12, 13, 14]. Thus far, only a few studies have evaluated cytokine and chemokine profiles in LGI1‐AE, often involving small sample sizes, limited analytes, and rarely interrogating clinical associations [15, 16, 17].

Similarly, neurofilament light chain (NfL), a biomarker of neuroaxonal damage, can offer important prognostic insight into disease activity and longitudinal outcomes in immune‐mediated neurological diseases [18, 19, 20, 21]. Thus far, studies evaluating the utility of NfL in the context of LGI1‐AE have been limited, primarily focusing on comparisons to other disease states [22, 23]. Whether cytokine/chemokine profiles and NfL levels can provide specific prognostic information in individuals with LGI1‐AE warrants further investigation. This study aims to identify serum and CSF cytokine/chemokine profiles in LGI1‐AE by evaluating 17 analytes associated with different arms of the immune response, and assess whether cytokine, chemokine, and/or NfL levels can offer prognostic information on risk of relapse, chronic epilepsy, and/or functional impairment at last follow‐up.

## 2 Methods

This retrospective observational study was approved by the Mayo Clinic Institutional Review Board (IRB #21‐000918); patients consented to having their records utilized for research.

### 2.1 Clinical Cohort

The LGI1‐AE cohort consisted of 44 individuals evaluated at a Mayo Clinic site (01/2015‐02/2024), with a positive serum and/or CSF LGI1‐IgG by cell‐based assay, a compatible phenotype, and available CSF for further testing. Patients with co‐existing neural antibodies were excluded. CSF samples were either frozen within two hours (prospective collection) or stored at 4°C for up to 30 days before freezing, possibly undergoing freeze–thaw cycles (retrospective collection). Paired sera within seven days of CSF collection were tested when available, if no immunotherapy was administered in the interim.

### 2.2 Comparator Cohorts

To establish our reference range, we used a mixed non‐inflammatory disorders (MNID) cohort of 42 patients with normal pressure hydrocephalus, idiopathic intracranial hypertension, or functional neurological disorders (34 previously published) [24]. All MNID samples were processed and stored at −20°C within two hours of collection until testing. To assess whether specific analyte elevations result from cellular changes related to cognitive decline/neurodegeneration or seizure activity independent of the underlying disease process, we also analyzed CSF and paired serum samples from 26 patients with Alzheimer's disease (AD) and seven patients with active idiopathic temporal lobe epilepsy (TLE; CSF collected within one week of a seizure). AD and TLE samples were stored at 4°C for up to 30 days before freezing. Individuals with CSF pleocytosis (> 5 cells/μL), comorbid malignancy, and/or systemic inflammatory disorders were excluded from all comparator groups (MNID, AD, TLE).

### 2.3 Lab‐Based LGI1‐IgG Seropositive Cohort

To support the analyte findings in the clinical cohort, a laboratory‐based cohort involving first‐time LGI1‐IgG positive patient samples in the Mayo Clinic neuroimmunology laboratory was included, more closely reflecting patients evaluated at the time of diagnosis outside of a tertiary referral center. The laboratory‐based cohort consisted of 55 CSF and available paired serum samples from patients with LGI1‐IgG detected by fixed cell‐based assay in serum and/or CSF in the Mayo Clinic Neuroimmunology Laboratory (05/2023–02/2024). There was no available clinical data for this cohort. Samples with co‐existing neural autoantibodies were excluded. All samples were stored at 4°C for up to 30 days before freezing.

### 2.4 Primary Clinical Outcomes

The primary outcomes evaluated were risk of relapse, chronic epilepsy at last follow‐up, and functional impairment at last follow‐up. Clinical relapse was defined as new or worsening symptoms after ≥ 1 month of clinical stability [25]. Chronic epilepsy was defined as the presence of ongoing seizures in the last six months before last follow‐up [26]. Functional impairment at last follow‐up was retrospectively assessed using the modified Rankin Score (mRS; patients with scores > 2 were considered severely impaired) [27] and the Clinical Assessment Scale in Autoimmune Encephalitis (CASE; scores ≥ 6 were considered severely impaired) [28].

### 2.5 Analyte Measurements

A multiplex immunoassay (ELLA by Bio‐Techne) was utilized to evaluate interleukin (IL)‐1‐beta, IL‐2, IL‐4, IL‐5, IL‐6, IL‐10, IL‐12p70, IL‐13, IL‐17A, B‐cell activating factor (BAFF), IL‐8/chemokine ligand CXCL8, CXCL9, CXCL10, CXCL13, granulocyte‐macrophage colony stimulating factor (GM‐CSF), interferon (IFN)‐gamma, tumor necrosis factor (TNF)‐alpha, and NfL, according to the manufacturer's instructions. Details on assay validation, resulting, and analyte thermal stability have been previously described [24].

### 2.6 Statistical Analysis

Analyte concentrations above the highest observed in MNID (or the lower limit of quantitation reported, whichever was higher) were considered elevated. Only analytes elevated in ≥ 15% of the LGI1‐AE patients were considered clinically relevant and were further assessed for clinical correlations. Wilcoxon's rank sum test was performed to compare analyte concentrations between groups. Due to differences in age across comparator cohorts, we elected not to perform a comparison of NfL levels across groups. A correction for multiple comparisons was not performed in order to preserve statistical power and minimize Type II errors. This approach was used to enable the detection of potentially meaningful effects, thereby facilitating hypothesis generation for future testing.

A univariate analysis was conducted to evaluate the associations between cytokine, chemokine, and NfL concentrations and clinical characteristics, utilizing Wilcoxon's rank sum test for continuous variables and Fisher's exact test for categorical variables. Multivariable logistic regression was performed to assess the associations between clinical characteristics and CSF analytes, with separate models for IL‐6 and IL‐8/CXCL8, and for NfL concentrations. The clinical outcome of interest served as the dependent variable, while sex, age at collection, immunotherapy at collection, time from onset to treatment, time from onset to CSF collection, and prospective collection of samples were included as potential covariates. Model selection was performed once for the IL‐6 and IL‐8/CXCL8 pair and separately for the NfL concentrations, using backward stepwise selection based on Bayesian Information Criterion (BIC), implemented in R via the stepAIC function from the MASS package with k = log (*n*) [29]. The range of models examined in the stepwise search was defined using formula specifications. The area under the curve (AUC) was calculated for each final model, and for the corresponding model excluding the analyte of interest to assess their predictive ability. A *p*‐value < 0.05 was considered statistically significant; missing values were excluded. The statistical software R was used for analysis [30].

## 3 Results

### 3.1 LGI1‐IgG‐Positive Patient Characteristics

The clinical cohort was comprised of 44 clinically confirmed LGI1‐AE patients included in the primary analysis; 14 (32%) had prospectively collected samples (stored frozen within two hours of collection), and 20 (45%) had paired sera available for analysis. Demographics and clinical characteristics of this cohort are outlined in Table 1. In the clinical cohort, most patients initially presented with seizures (39, 89%), of whom 18 (41%) had faciobrachial dystonic seizures (FBDS); 22 patients (50%) had cognitive impairment upon presentation. Four (9%) had a new or recurrent malignancy discovered within 2 years of their LGI1‐AE diagnosis [31], including melanoma (*n* = 2), prostate adenocarcinoma (*n* = 1), and appendiceal mucinous carcinoma (*n* = 1). Twelve (27%) had a comorbid autoimmune disease, including hypothyroidism (*n* = 8), vitiligo (*n* = 1), psoriasis/psoriatic arthritis (with adequate disease control on etanercept) (*n* = 1), autoimmune hepatitis (*n* = 1), and perinuclear antineutrophil cytoplasmic antibody (p‐ANCA)‐associated vasculitis (lung‐restricted, stable for 2 years off azathioprine) (*n* = 1).

**TABLE 1:** Descriptive characteristics, diagnostic testing, treatment, and outcomes of clinical LGI1‐IgG cohort (*N* = 44).

Demographics	
Male sex, *n* (%)	29 (66)
Age at diagnosis, median (range)	68.5 (8–85)
Age at collection, median (range)	69.5 (8–85)
Presenting clinical features	
Seizures, *n* (%)	39 (89)
FBDS, *n* (%)	18 (41)
Cognitive impairment, *n* (%)	22 (50)
Psychiatric disturbance, *n* (%)	7 (16)
Paraneoplastic^a^, *n* (%)	4 (9)
Non‐neurological autoimmune disease, *n* (%)	12 (27)
mRS at diagnosis, median (IQR)	2 (2–3)
CASE at diagnosis, median (IQR)	4 (3–5)
Diagnostic testing	
Onset to CSF collection, months, median (IQR)	8 (3–17)
Prospective sample collection, *n* (%)	14 (32)
Immunotherapy at CSF collection, *n* (%)	12 (27)
CSF pleocytosis, *n* (%)	2 (5)
CSF WBC count, median (range)	1 (0–7)
MRI temporal lobe T2/FLAIR change^b^, *n* (%)	25 (57)
MRI temporal lobe atrophy^b^, *n* (%)	13/30 (43)
Immunosuppressive/modulating treatment	
Onset to treatment, months, median (IQR)	5 (3–8)
Immunosuppressive/modulating therapy, *n* (%)	40 (91)
Acute, Steroids, *n* (%)	31/40 (78)
Acute, IVIG, *n* (%)	15/40 (38)
Acute, Plasmapheresis, *n* (%)	5/40 (13)
Maintenance^c^, Corticosteroids, *n* (%)	26/40 (65)
Maintenance^c^, IVIG, *n* (%)	6/40 (15)
Maintenance^c^, Mycophenolate, *n* (%)	9/40 (23)
Maintenance^c^, Azathioprine, *n* (%)	1/40 (3)
Maintenance^c^, Rituximab, *n* (%)	10/40 (25)
Clinical outcomes	
Onset to last visit, months, median (IQR)	22 (14–37)
Any clinical relapse^d^, *n* (%)	19/42 (45)
Clinical relapse after CSF collection, *n* (%)	14/42 (33)
mRS at last visit, median (IQR)	2 (1–2)
CASE at last visit, median (IQR)	3 (1.75–4)
CASE, seizure sub‐scale, median (IQR)	1 (0–1.25)
CASE, memory sub‐scale, median (IQR)	1 (0–1.25)
Chronic epilepsy^e^ at last visit, *n* (%)	12 (27)
Prescribed ASM at last visit, *n* (%)	28 (64)

Abbreviations: ASM, anti‐seizure medication; CASE, clinical assessment scale in autoimmune encephalitis; CSF, cerebrospinal fluid; FBDS, faciobrachial dystonic seizures; IVIG, intravenous immune globulin; mRS, modified Rankin Scale; WBC, white blood cell.

^a^ New or recurrent malignancy identified within 2 years of diagnosis.

^b^ At any timepoint during follow‐up.

^c^ Treatment for at least 3 months.

^d^ New or worsening neuropsychiatric symptoms after at least 1 month of clinical stability.

^e^ Persistent seizures despite treatment with antiseizure medication.

The median time from symptom onset to CSF collection was eight months (IQR, 3–17). Only eight (18%) samples were collected within three months of symptom onset. Twelve (27%) patients were receiving immunotherapy at CSF collection (including 7/14 [50%] with prospectively collected samples); a total of 13 (30%) received immunotherapy at any time before/at CSF collection. Most (40, 91%) received immunotherapy during their disease course, with a median time from symptom onset to immunotherapy initiation of five months (IQR, 3–8). The median follow‐up time was 22 months (IQR, 14–36) from symptom onset. Less than half of the patients with available longitudinal follow‐up sustained a clinical relapse (19/42, 45%), many were on an anti‐seizure medication (ASM) at the last visit (28, 64%), and a minority (12, 27%) developed chronic epilepsy.

The lab‐based LGI1‐IgG‐positive cohort included 58 unique patient CSF samples (stored at 4°C for up to 30 days before freezing) with 30 (52%) paired serum samples. The total combined LGI1‐AE cohort consisted of both the clinical and lab‐based cohorts, resulting in 102 unique LGI1‐AE CSF samples for cytokine, chemokine, and NfL analysis (Table S1). The lab‐based cohort had a median age of 68 (range 16–86) and 29 (50%) were male. Demographic and analyte comparisons between the combined LGI1‐AE cohort (clinical and lab‐based) and control cohorts in CSF and serum are outlined in Tables 2 and 3, respectively. Independent analyte comparisons between MNID and the clinical and laboratory‐based LGI1‐AE cohorts are presented in Table S1.

**TABLE 2:** Comparison of demographics and CSF analyte levels between the total LGI1‐AE cohort and noninflammatory, AD, and TLE controls.

	Total LGI1‐AE (*N* = 102)		MNID (*N* = 42)	*p* ^a^	Alzheimer's disease (*N* = 26)		*p* ^a^	Temporal lobe epilepsy (*N* = 7)		*p* ^a^
Male sex, *n* (%)	58 (57)		25 (60)	0.85	13 (50)		0.66	3 (43)		0.70
Median age at collection, years (range)	68 (8–86)		74 (21–87)	0.005*	60.5 (52–82)		0.008*	63 (22–77)		0.07
CSF Analyte	Median, pg/mL (IQR)	Elevated (%)	Median, pg/mL (IQR)		Median, pg/mL (IQR)	Elevated (%)		Median, pg/mL (IQR)	Elevated (%)	
IL‐1‐beta	0.2 (0.2, 0.4)	4/99 (4)	0.3 (0.2, 0.4)	n/a^b^	0.1 (0, 0.1)	1/23 (4)	n/a^b^	0.4 (0.4, 0.4)	0/6 (0)	n/a^b^
IL‐2	0.2 (0.1, 0.3)	4/102 (4)	0.2 (0.2, 0.3)	n/a^b^	0.2 (0.1, 0.3)	2/23 (9)	n/a^b^	0.1 (0.1, 0.1)	0/7 (0)	n/a^b^
IL‐4	0 (0, 0)	2/101 (2)	0 (0,0.1)	n/a^b^	0.2 (0.1, 0.2)	1/22 (5)	n/a^b^	0 (0, 0.1)	0/7 (0)	n/a^b^
IL‐5	0.3 (0.2,0.5)	2/102 (2)	0.3 (0.2, 0.4)	n/a^b^	0.3 (0.2, 0.4)	1/26 (4)	n/a^b^	0.4 (0.3, 0.4)	0/7 (0)	n/a^b^
IL‐6	3.5 (2.3,9.1)	25/100 (25)	3.2 (2.5, 4.9)	0.43	2.2 (1.8, 4)	3/23 (13)	0.06	2.8 (2.3, 9.3)	2/7 (29)	0.82
IL‐10	0.6 (0.4, 1.0)	9/100 (9)	0.6 (0.4, 0.7)	n/a^b^	0.3 (0.2, 0.4)	2/21 (10)	n/a^b^	0.8 (0.6, 1.1)	0/6 (0)	n/a^b^
IL‐12p70	0.3 (0.2, 0.4)	0/98 (0)	0.4 (0.2, 0.5)	n/a^b^	0.1 (0, 0.1)	1/20 (5)	n/a^b^	0.5 (0.4,0.5)	0/7 (0)	n/a^b^
IL‐13	0 (0, 0)	3/101 (3)	0 (0, 0)	n/a^b^	0 (0, 0)	1/22 (5)	n/a^b^	0 (0, 0)	0/7 (0)	n/a^b^
IL‐17A	1.2 (0.7, 1.8)	22/94 (23)	0.7 (0.3, 1.1)	< 0.001*	1 (0.6, 1.6)	2/20 (10)	0.45	0.5 (0.4, 1.1)	1/7 (14)	0.11
BAFF	145 (100, 226)	11/102 (11)	118 (94, 157)	n/a^b^	111 (84, 157)	1/26 (4)	n/a^b^	144 (120, 167)	0/7 (0)	n/a^b^
IL‐8/CXCL8	47 (31, 93)	18/102 (18)	33 (26, 42)	< 0.001*	38 (28, 44)	0/26 (0)	0.01*	55 (41, 85)	1/7 (14)	0.62
CXCL9	58 (30, 118)	5/102 (5)	75 (56, 132)	n/a^b^	37 (23, 75)	1/26 (4)	n/a^b^	16 (9, 79)	0/7 (0)	n/a^b^
CXCL10	149 (62, 283)	9/102 (9)	185 (144, 255)	n/a^b^	108 (53, 160)	0/26 (0)	n/a^b^	67 (49, 133)	0/7 (0)	n/a^b^
CXCL13	3.0 (1.6, 6.4)	5/101 (5)	3.4 (2.2, 5.0)	n/a^b^	2.3 (1.5, 3.2)	0/26 (0)	n/a^b^	2.2 (1.7, 4.4)	1/7 (14)	n/a^b^
GM‐CSF	0.1 (0, 0.4)	9/100 (9)	0.4 (0.2, 0.5)	n/a^b^	0.1 (0, 0.1)	1/26 (4)	n/a^b^	0.7 (0.4, 0.9)	2/7 (29)	n/a^b^
IFN‐gamma	0 (0, 0.1)	7/75 (9)	0 (0, 0.1)	n/a^b^	0 (0, 0)	1/21 (5)	n/a^b^	0 (0, 0.1)	0/7 (0)	n/a^b^
TNF‐alpha	0.8 (0.5, 1.1)	4/98 (4)	0.9 (0.7, 1.1)	n/a^b^	0.6 (0.5, 0.9)	1/26 (4)	n/a^b^	0.8 (0.6, 0.8)	0/7 (0)	n/a^b^

*Note:* Mixed non‐inflammatory disease cohort: normal pressure hydrocephalus [32], idiopathic intracranial hypertension [2], functional neurologic disease [2], and other neurologic disease [3].

Abbreviations: BAFF, B‐cell activating factor; CXCL, chemokine ligand; GM‐CSF, granulocyte‐macrophage colony stimulating factor; IFN, interferon; IL, interleukin; TNF, tumor necrosis factor.

^a^ Wilcoxon rank sum test.

^b^ Analyte elevated in < 15% of LGI1‐AE patients.

* Statistically significant (*p* < 0.05).

**TABLE 3:** Comparison of demographics and serum analyte levels between the total LGI1‐AE cohort and non‐inflammatory, AD, and TLE controls.

	Total LGI1‐AE (*N* = 49)		MNID (*N* = 11)	*p* ^a^	Alzheimer's disease (*N* = 13)		*p* ^a^	Temporal lobe epilepsy (*N* = 8)		*p* ^a^
Male sex, *n* (%)	26 (53)		4 (36)	0.51	9 (69)		0.36	3 (38)		0.47
Median age at collection, years (range)	68 (8–83)		31 (23–49)	< 0.001*	56 (52–82)		0.009*	46 (15–64)		< 0.001*
Serum analyte	Median, pg/mL (IQR)	Elevated (%)	Median, pg/mL (IQR)		Median, pg/mL (IQR)	Elevated (%)		Median, pg/mL (IQR)	Elevated (%)	
IL‐1‐beta	0.1 (0.1, 0.3)	22/49 (45)	0 (0, 0)	< 0.001*, ^c^	0.2 (0.1, 0.4)	9/13 (69)	0.42	0.1 (0, 0.1)	1/8 (13)	0.02*, ^c^
IL‐2	0.1 (0, 0.2)	1/49 (2)	0 (0, 0.1)	n/a^b^	0.1 (0, 0.3)	0/13 (0)	n/a^b^	0.1 (0, 0.3)	0/8 (0)	n/a^b^
IL‐4	0 (0, 0)	2/49 (4)	0 (0, 0)	n/a^b^	0 (0, 0)	1/13 (8)	n/a^b^	0 (0, 0)	0/7 (0)	n/a^b^
IL‐5	0.4 (0.2, 0.6)	9/49 (18)	0.3 (0.2, 0.3)	0.10	0.3 (0.2, 0.7)	4/13 (31)	0.72	0.4 (0.3, 0.8)	3/8 (38)	0.57
IL‐6	5.2 (3, 12)	41/48 (85)	1.2 (1.1, 1.8)	< 0.001*	2.8 (1.9, 4.4)	8/13 (62)	0.04*	4.9 (2.5, 5.9)	6/8 (75)	0.37
IL‐10	1.3 (1, 2)	3/48 (6)	1 (0.9, 1.5)	n/a^b^	1.1 (0.7, 1.3)	0/13 (0)	n/a^b^	1.3 (1.1, 1.8)	0/8 (0)	n/a^b^
IL‐12p70	0.5 (0.3, 0.9)	19/47 (40)	0 (0, 0.6)	0.02* ^,c^	0.6 (0.5, 1.1)	6/13 (46)	0.19	0.6 (0.4, 0.6)	2/8 (25)	0.91
IL‐13	0 (0, 1.3)	2/48 (4)	2 (0, 4.7)	n/a^b^	2.1 (0.4, 6.2)	0/13 (0)	n/a^b^	2.2 (0.2, 10)	0/8 (0)	n/a^b^
IL‐17A	1.3 (0.7, 1.9)	26/46 (57)	0.2 (0, 0.2)	< 0.001*	2.3 (1.8, 5.4)	10/10 (100)	0.004*	0.8 (0.5, 1.2)	4/8 (50)	0.09
BAFF	453 (341, 583)	7/49 (14)	518 (413, 586)	n/a^b^	587 (551, 820)	5/13 (39)	n/a^b^	596 (453, 675)	1/8 (13)	n/a^b^
IL‐8/CXCL8	17 (13, 30)	19/49 (39)	11 (10, 15)	0.03*	19 (17, 26)	6/13 (46)	0.47	14 (12, 16)	0/8 (0)	0.18
CXCL9	648 (357, 1058)	13/49 (27)	274 (232, 482)	0.04*	523 (399, 758)	2/12 (17)	0.77	491 (335, 602)	1/8 (13)	0.35
CXCL10	98 (49, 158)	2/49 (4)	100 (76, 151)	n/a^a^	77 (31, 113)	0/13 (0)	n/a^b^	60 (43, 71)	0/8 (0)	n/a^b^
CXCL13	25 (13, 41)	0/49 (0)	59 (54, 80)	n/a^b^	19 (9, 23)	0/13 (0)	n/a^b^	15 (12, 37)	0/8 (0)	n/a^b^
GM‐CSF	0.5 (0.2, 1)	12/48 (25)	0 (0, 0.1)	< 0.001*	0.8 (0.6, 2.7)	6/13 (46)	0.01*	0.8 (0.5, 1.7)	3/8 (38)	0.20
IFN‐gamma	0.5 (0.3, 1.5)	7/49 (14)	0.4 (0.3, 0.8)	n/a^b^	0.8 (0.5, 1.2)	2/13 (15)	n/a^b^	0.6 (0.4, 0.8)	0/8 (0)	n/a^b^
TNF‐alpha	6.9 (5.3, 10.1)	4/49 (8)	10.4 (8.5, 12.4)	n/a^b^	8 (5.1, 9.3)	0/13 (0)	n/a^b^	7.1 (5.7, 8.1)	0/8 (0)	n/a^b^

*Note:* Mixed non‐inflammatory disease cohort: healthy individuals [8], idiopathic intracranial hypertension [3].

Abbreviations: BAFF, B‐cell activating factor; CXCL, chemokine ligand; GM‐CSF, granulocyte‐macrophage colony stimulating factor; IFN, interferon; IL, interleukin; TNF, tumor necrosis factor.

^a^ Wilcoxon rank sum test.

^b^ Analyte elevated in < 15% of LGI1‐AE patients.

^c^ Median values below the lower limit of quantitation of the assay.

* Statistically significant (*p* < 0.05).

### 3.2 LGI1‐AE Serum and CSF Analytes Versus Mixed Non‐Inflammatory Disease (MNID) Controls

**FIGURE 1:**
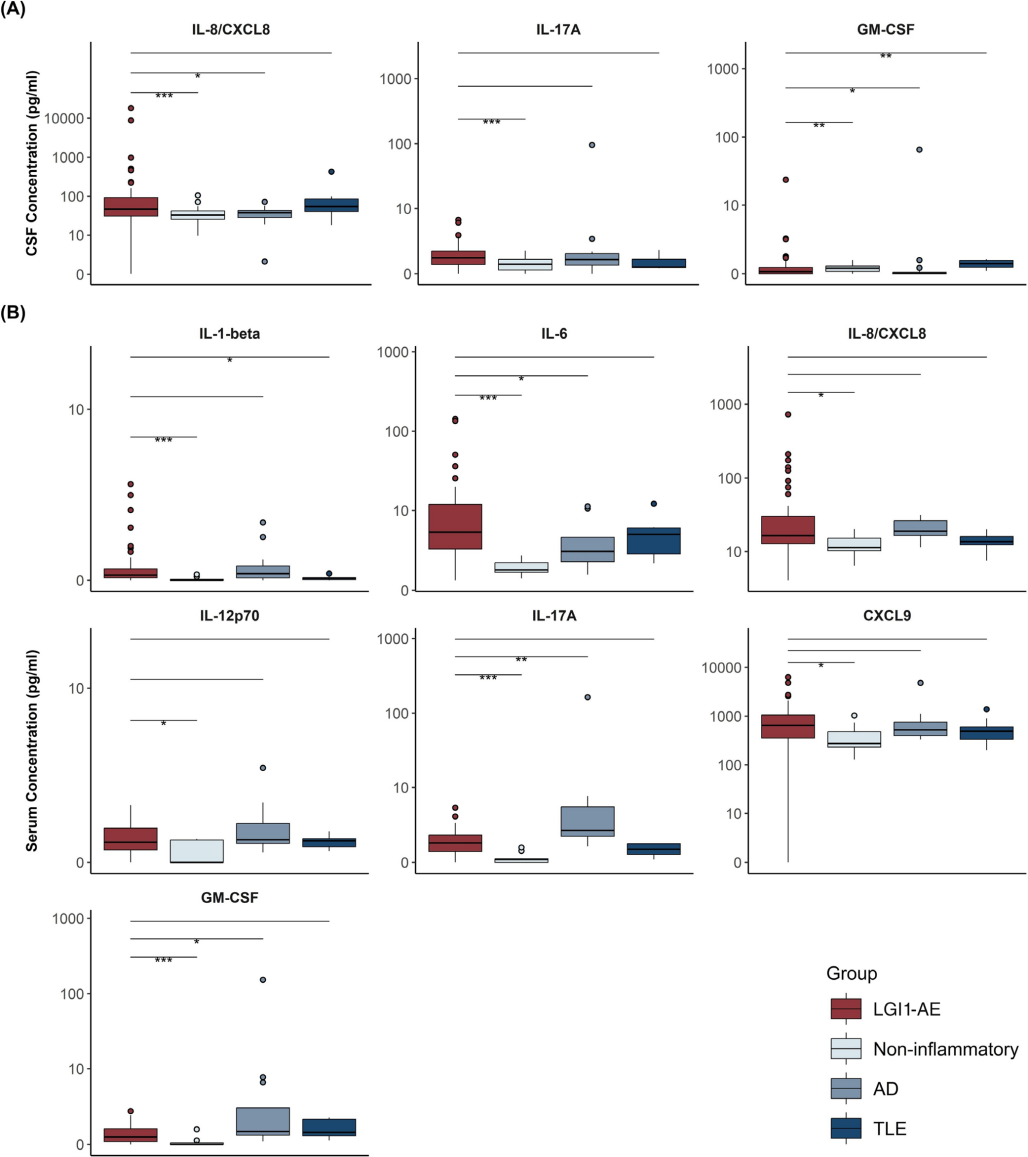
Cerebrospinal fluid (A) and serum (B) cytokine and chemokine concentrations in total LGI1‐AE and controls. Only boxplots of significantly elevated analytes in LGI1‐AE in comparison to non‐inflammatory controls are presented. Significant differences between LGI1‐AE and individual control groups (Wilcoxon's rank‐sum test) are indicated by asterisks (*p < 0.05; **p < 0.01; ***p < 0.001).

**FIGURE 2:**
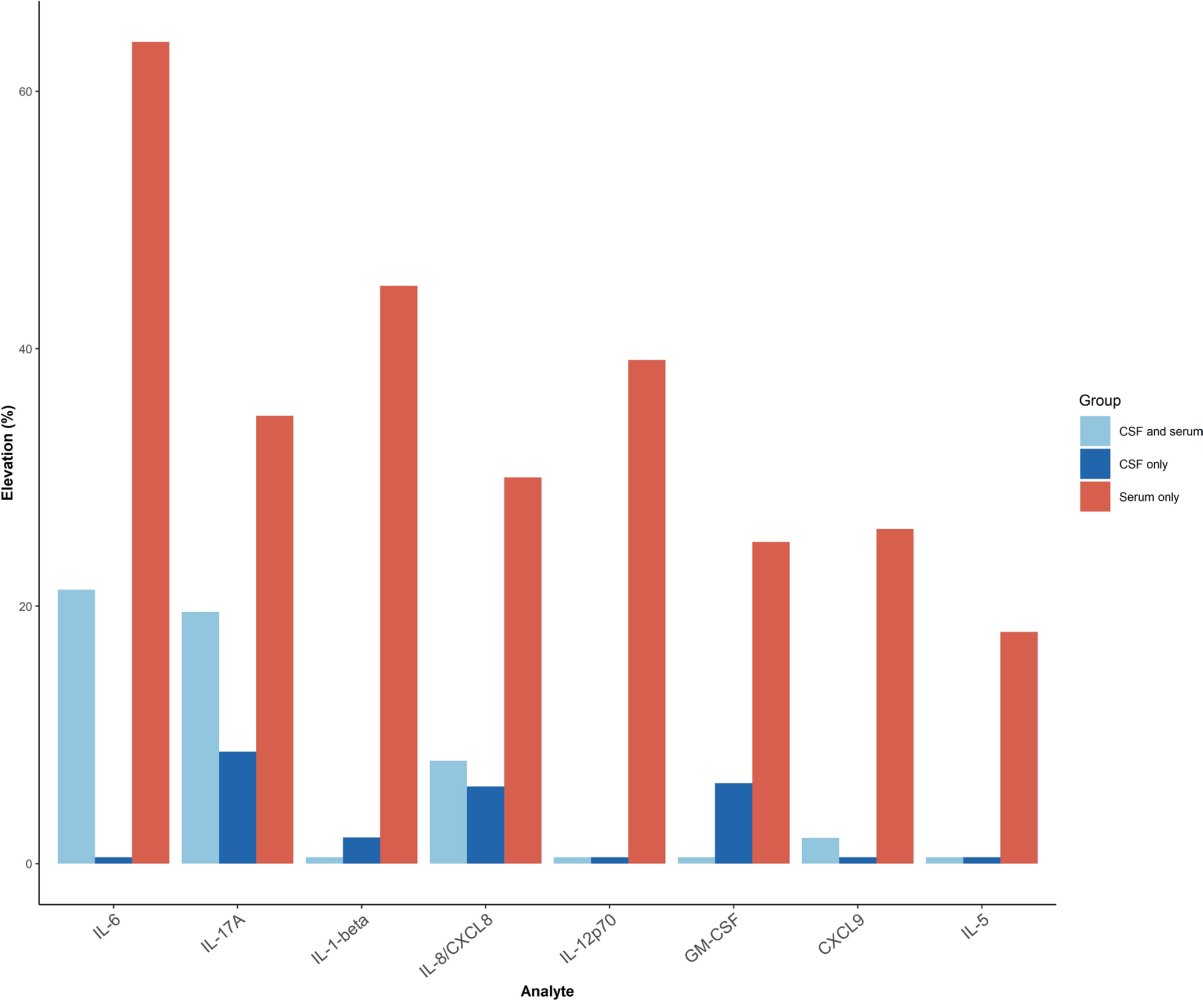
Percentage of cytokine/chemokine elevations per specimen type in total LGI1‐AE cohort with paired serum and cerebrospinal fluid (CSF) (*N* = 50). Only analytes elevated in at least 15% of the cohort were included. IL, interleukin.

**FIGURE 3:**
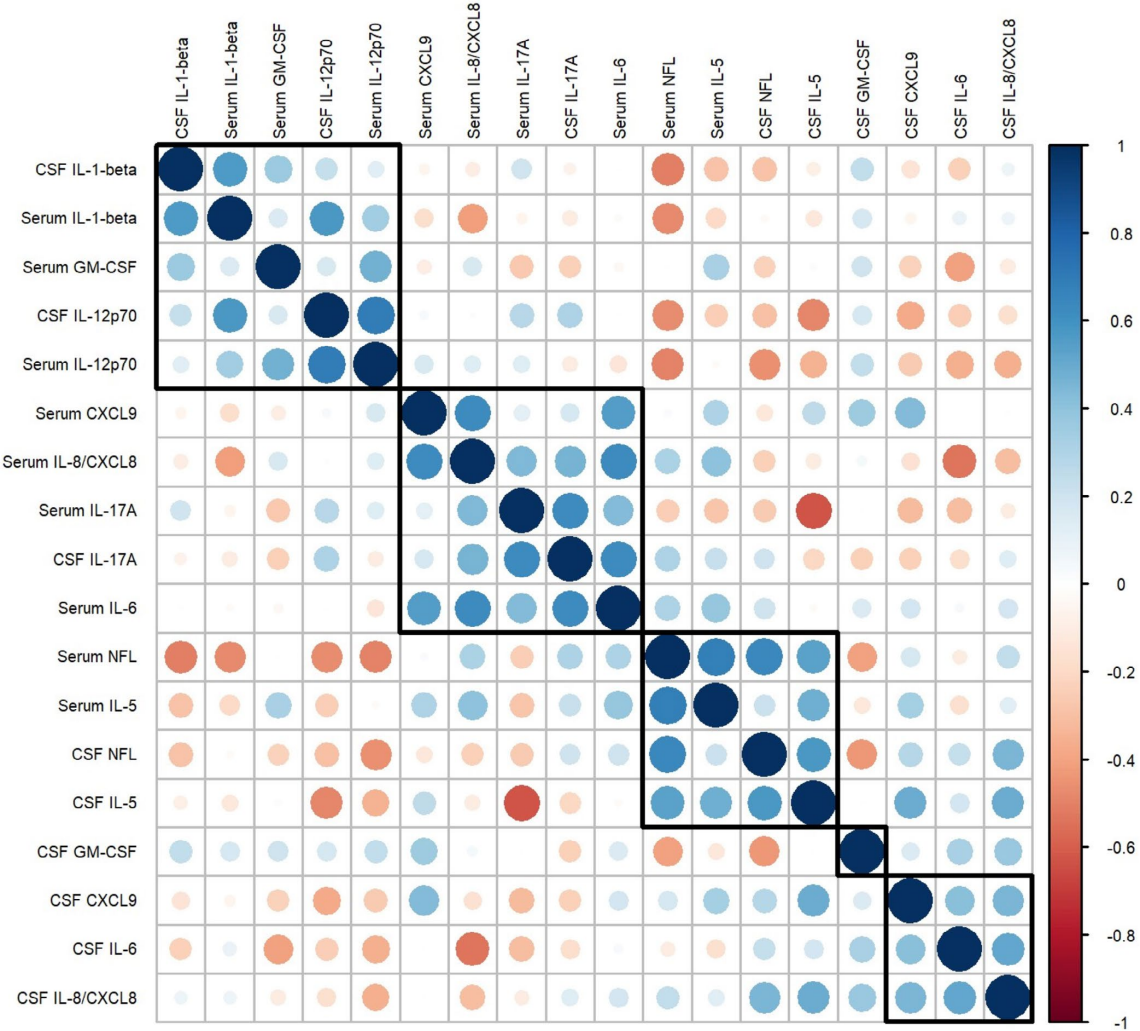
Correlation and clustering of serum and cerebrospinal fluid analytes in individual clinical LGI1‐AE patients. Correlogram of Spearman's rank correlation coefficient matrix with hierarchical clustering of analytes. Only analytes elevated in at least 15% of the cohort were included. IL = interleukin.

Compared to the MNID cohort, the combined LGI1‐AE cohort had higher concentrations of serum and CSF IL‐8/CXCL8 (*p* = 0.03 and *p* < 0.001 respectively) and IL‐17A (*p* < 0.001 for both), and higher serum IL‐6 (*p* < 0.001), CXCL9 (*p* = 0.04), and GM‐CSF (*p* < 0.001) (Tables 2 and 3, Figure 1). Serum IL‐1‐beta and IL‐12p70 levels were also higher compared to MNID, but median levels were below the lower limit of quantitation for the assay. When separately compared to MNID, the laboratory‐based cohort additionally had higher levels of CSF IL‐6 (*p* = 0.007), IL‐10 (*p* = 0.007), and BAFF (*p* < 0.001), and all three of these analytes were elevated in ≥ 15% of patients in the laboratory‐based cohort (Table S1). The most frequently elevated analytes in the combined LGI1‐AE cohort were IL‐6 (85%), IL‐17A (57%), IL‐1‐beta (45%), IL‐12p70 (40%), IL‐8/CXCL8 (39%), CXCL9 (27%), GM‐CSF (25%), and IL‐5 (18%) in the serum, and IL‐6 (25%), IL‐17A (23%), and IL‐8/CXCL8 (18%) in the CSF. Serum analytes were more commonly elevated than CSF analytes, and percentage elevation was analyte‐dependent (Figure 2). CSF IL‐6 and IL‐8/CXCL8 tended to cluster together, as did serum and CSF IL‐17A, serum IL‐6, IL‐8/CXCL8, and CXCL9 (Figure 3).

Analyte concentrations of prospectively collected LGI1‐AE samples (frozen within two hours of collection) were higher for serum IL‐10 and CXCL13, and CSF IL‐10, CXCL10, and TNF‐alpha compared to retrospectively collected samples (Table S2).

### 3.3 LGI1‐AE Serum and CSF Analytes Versus Disease Controls

As compared to AD (Figure 1; Tables 2 and 3), the combined LGI1‐AE cohort had higher levels of serum IL‐6 (*p* = 0.04) and CSF IL‐8/CXCL8 (*p* = 0.01). The laboratory‐based LGI1‐AE cohort had higher levels of both serum and CSF IL‐6 (*p* = 0.03; *p* = 0.002), in addition to higher CSF IL‐8/CXCL8 (*p* < 0.001). Conversely, AD controls demonstrated higher levels of serum IL‐17A (*p* = 0.004) and GM‐CSF (*p* = 0.01) compared with the combined LGI1‐AE cohort. Notably, serum IL‐17A was elevated in all AD controls.

No cytokines/chemokines were elevated in the combined LGI1‐AE cohort compared to the active TLE cohort (Figure 1; Tables 2 and 3). The laboratory‐based cohort had higher levels of serum and CSF IL‐17A (*p* = 0.01 and *p* = 0.02 respectively) while TLE had higher levels of CSF GM‐CSF (*p* = 0.03). IL‐6 levels were similar in both serum and CSF between the two cohorts.

### 3.4 Association Between Analytes and Clinical Outcomes

**FIGURE 4:**
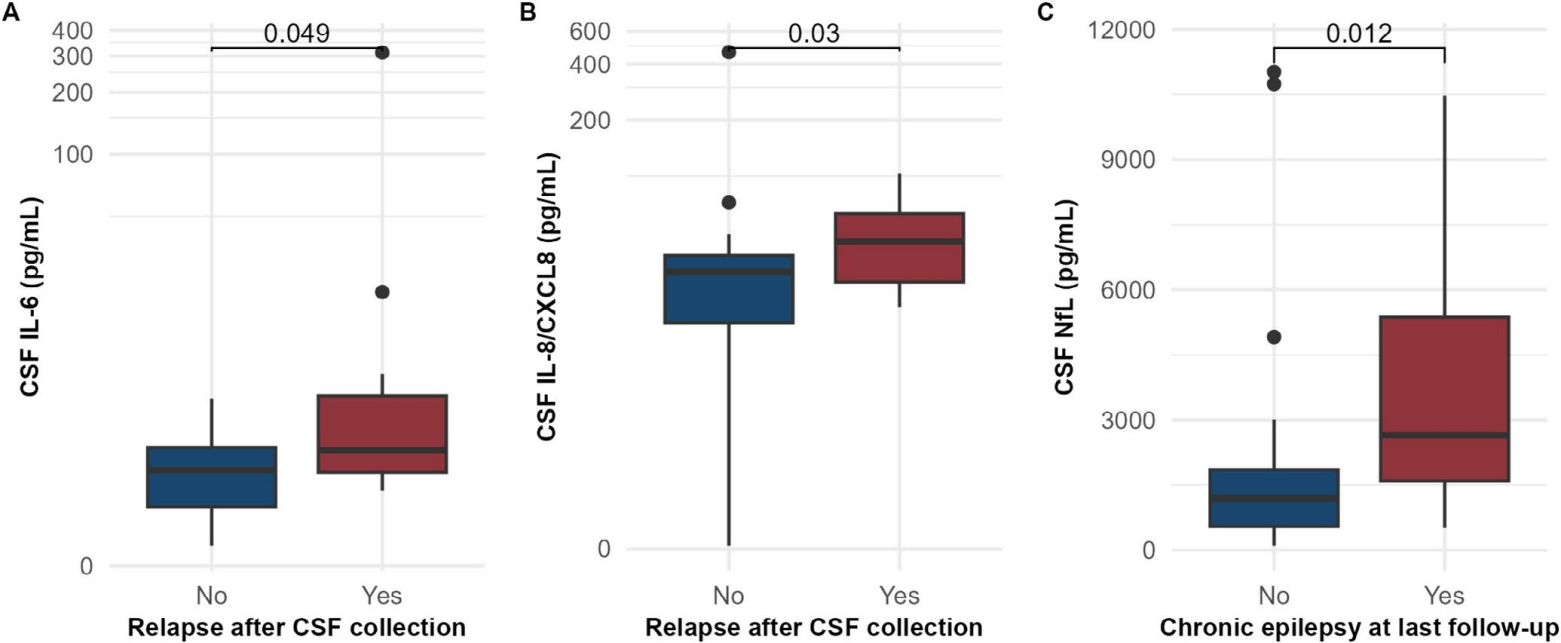
Analyte associations with longitudinal outcome data. (A) Boxplot of CSF IL‐6 levels in patients with and without relapse after CSF collection. (B) Boxplot of CSF IL‐8/CXCL8 in patients with and without relapse after CSF collection. (C) Boxplot of neurofilament light chain (NfL) levels in patients with and without chronic epilepsy at last follow‐up. Comparisons were performed using Wilcoxon's rank sum test.

In the univariate analysis, having at least one clinical relapse at any time between symptom onset to last follow‐up was associated with higher concentrations of CSF IL‐6 (*p* = 0.02) and/or CSF IL‐8/CXCL8 (*p* = 0.04) at the time of collection (Figure 4). Similarly, higher CSF IL‐6 and CSF IL‐8/CXCL8 were also associated with a higher relapse rate after CSF collection (*p* < 0.05). In the multivariable analysis, CSF IL‐6 was the only variable retained in the final model and remained predictive of a relapsing course both throughout the disease course and after CSF collection (2.9‐ and 2.6‐fold increase in the odds of relapse for each two‐fold increase in analyte concentration, respectively; *p* ≤ 0.03). The addition of CSF IL‐6 in the model significantly increased the AUC (0.71 vs. 0.50, 0.009) for predicting a relapsing course (Table S3).

CSF NfL levels were positively associated with chronic epilepsy (*p* = 0.01) at last follow‐up (Figure 4). Age was similar between the groups compared. CSF NfL and sex were retained in the final model for predicting chronic epilepsy. A two‐fold increase in CSF NfL concentration was associated with a 1.9‐fold higher odds of developing chronic epilepsy (*p* = 0.03). Male sex was also independently predictive of chronic epilepsy (OR = 10.3; *p* < 0.05). The addition of CSF NfL in the model increased the AUC (0.82 vs. 0.68; *p* = 0.01) for predicting chronic epilepsy (Table S3). No associations were found between cytokine, chemokine, or NfL levels and functional impairment (mRS, CASE).

## 4 Discussion

This study evaluated the cytokine, chemokine, and NfL concentrations in patients with LGI1‐AE, demonstrating elevations in IL‐6, IL‐8/CXCL8, and IL‐17A compared to controls. Specifically, elevations in IL‐6 and IL‐8/CXCL8 appeared to cluster together in patients. Furthermore, IL‐6 and IL‐8/CXCL8 levels at CSF collection were associated with the risk of relapse, while elevated NfL was associated with chronic epilepsy at the last follow‐up. There was no association between the evaluated cytokines/chemokines or NfL and longitudinal functional impairment. Taken together, these findings inform the underlying pathophysiology of LGI1‐AE, and offer prognostic insight into the clinical course, although larger prospective studies are needed to confirm these exploratory findings.

The cytokine/chemokine signature demonstrated in LGI1‐AE appears to involve primary elevations in IL‐17A, IL‐6, and IL‐8/CXCL8. Elevations in IL‐6 have previously been identified in several immune‐mediated neurologic diseases, most notably neuromyelitis optica spectrum disorder (NMOSD), resulting in novel therapeutic strategies highly effective in controlling the disease [10, 33]. Our findings suggest that IL‐6 may play a role in the pathophysiology of LGI1‐AE. A clinical trial is currently underway to further investigate IL‐6 as a potential therapeutic target (NCT05503264) [34].

Notably, cytokine/chemokine levels, including IL‐6, were comparable between the active TLE and LGI1‐AE cohorts. Elevated serum IL‐6 levels are reported in patients experiencing acute seizures, particularly in those with active temporal lobe seizures [32, 35, 36, 37]. Elevated IL‐6 may reflect the inflammatory milieu contributing to heightened seizure frequency in LGI1‐AE or may be a consequence of the seizures themselves. The association between IL‐6 and seizures may offer one potential explanation as to why seizure activity in LGI1‐AE is particularly immunotherapy responsive and may support seizure activity as an important outcome measure in LGI1‐AE treatment trials [38].

Both serum and CSF IL‐17A were elevated in the LGI1‐AE cohort compared with the MNID cohort. IL‐17A has previously been implicated in neurological autoimmunity, including in LGI1‐AE, with CSF IL‐17A levels previously shown to correlate with disease severity in the acute phase [39]. While IL‐17A inhibitors have demonstrated efficacy in the treatment of psoriasis, they have not yet been explored in neurological autoimmunity beyond multiple sclerosis and may also warrant future investigation [40]. Interestingly, serum IL‐17A was elevated in all patients with AD, corroborating findings in previous studies [41]. Prior animal models have supported the role of IL‐17A in AD pathogenesis, including neuroinflammation, neurodegeneration, and progressive cognitive deficits [42].

CSF IL‐8/CXCL8 is primarily secreted by monocytes, macrophages, epithelial, and endothelial cells, playing a role in neutrophil chemotaxis underlying the response to infection and tissue injury [43]. Like several other cytokines and chemokines, IL8/CXCL8 is also produced by neural cells [44, 45]. IL8/CXCL8 has been implicated in multiple diseases such as multiple sclerosis, glial fibrillary acidic protein autoimmunity, and infectious or post‐infectious syndromes including SARS‐CoV‐2 infection [43, 46, 47]. While likely not specific to AE, elevations in CSF IL‐8/CXCL8 may suggest inflammatory disease activity in LGI1‐AE, as evidenced by its association with relapse activity in this cohort.

One prior study identified CSF CXCL13 as a signature of LGI1‐AE [15]. While we did not identify an elevation in CSF CXCL13 within our cohort, we did identify an elevation in CSF BAFF—another B‐cell related chemokine—in our laboratory‐based cohort, which may further support the established role of B‐cells and antibody‐secreting cells in LGI1‐AE [48]. This elevation was not present in our clinical cohort and thus no clinical correlations were possible.

Cytokine and chemokine elevations in our LGI1‐AE cohort provided prognostic information. Increased levels of CSF IL‐6 and/or CSF IL‐8/CXCL8 at CSF collection predicted an increased risk of future clinical relapse. Given the absence of a standardized treatment approach for LGI1‐AE, and clinical equipoise regarding the benefit of maintenance immunotherapy, cytokine/chemokine profiling may help to identify patients at greater risk of relapse who could benefit from maintenance immunotherapy. This will need to be evaluated in larger prospective studies.

Furthermore, increased CSF NfL levels at collection were associated with the development of chronic epilepsy at last follow‐up. This was confirmed in a multivariate logistic regression model that included age as a variable. This finding aligns with a prior study which demonstrated that a longitudinal increase in NfL levels correlated with disease activity in AE (including LGI1‐AE), suggesting NfL as a surrogate for ongoing axonal damage [21]. The observed association between chronic epilepsy and CSF NfL in our study suggests that chronic epilepsy in LGI1‐AE may potentially be a complication of neurodegeneration [22]. Thus, CSF NfL may function as a prognostic marker of chronic epilepsy among patients with LGI1‐AE, a complication that can affect up to 20% of patients, and may have long‐term consequences on functional independence [26].

Neither mRS nor CASE associated with any elevations in cytokine, chemokine, or NfL. Previous studies evaluating the prognostic value of NfL as a predictor of functional outcomes in AE are limited, with most studies focusing on N‐methyl‐D‐aspartate receptor (NMDAR)‐AE [20, 23]. In one study, CSF NfL associated with a higher mRS score at last follow‐up in both LGI1‐AE and NMDAR‐AE [23]. We were not able to replicate these findings in our study. While cytokines, chemokines, and NfL did associate with key clinical outcomes in LGI1‐AE, the lack of an association with mRS and/or CASE may be due to the low sensitivity of these scores in measuring disease‐specific functional impairment in LGI1‐AE, or could be related to our relatively small clinical cohort and the nature of our clinical practice (tertiary referral center) [27, 28]. Future studies evaluating the prognostic utility of cytokine, chemokine, and NfL in predicting LGI1‐AE outcomes should involve LGI1‐AE specific outcomes scales [21, 49, 50].

There are several strengths of this study, including the application of a validated and sensitive assay for CSF testing to a large‐scale clinical cohort of LGI1‐AE patients, the inclusion of the lab‐based cohort, and the comparison to controls representing disease mimics. Prior studies investigating cytokine/chemokine profiles in LGI1‐AE have featured limited sample sizes (< 20 patients), with conflicting results [15, 16, 17]. The utilization of a large cohort involving more than 100 LGI1‐IgG positive samples in this study provides further clarity toward the cytokine/chemokine profile of patients with LGI1‐AE, contributing insight into the underlying pathophysiology of the disease.

There are also several limitations associated with this study, including its reliance on retrospective data collection, with most patients initially presenting outside of Mayo Clinic. Thus, several patients were evaluated at our center later in their disease course, with a longer interval between symptom onset and CSF collection. This delay also resulted in a proportion of patients (27%) receiving immunotherapy at CSF collection, affecting analyte concentrations. These factors likely affected cytokine levels and led to the differences observed between the clinical and laboratory‐based cohort. The clinical cohort was also subject to referral bias since patients were often referred for second opinion due to more severe or refractory disease. This may explain why our cohort had a higher rate of relapse and chronic epilepsy than previously reported [51], although relapse rates were similar to those reported in prior studies originating at our institution [26]. A small percentage of patients had non‐neurologic autoimmune disorders possibly affecting analyte concentration in the blood; most were related to presumed thyroid autoimmunity. The retrospective clinical cohort samples analyzed were also subject to repeat testing which may have affected cytokine stability; this was also possible for the laboratory‐based cohort, but we still observed clear analyte elevations in serum and CSF.

Additionally, when comparing retrospectively and prospectively collected samples, there were differences between analytes, although none were significantly elevated in comparison to the control groups nor associated with clinical outcomes. We have previously demonstrated that analyte stability is superior in the CSF compared to serum, with most sampled analytes remaining stable (< 30% loss) for up to 30 days at 4°C and/or five freeze–thaw cycles (except for IL‐1‐beta, IL‐2, and IL‐10) [24]. Furthermore, there were age differences between the LGI1‐AE and control cohorts (including the ones used to establish normal values), limiting our ability to perform NfL comparisons across groups. Regardless, NfL levels were evaluated for clinical correlations in a multivariate logistic regression model including age, and clinical associations persisted. While it is possible that some cases in the laboratory‐based cohort were false positives, this is very rare with LGI1‐IgG testing [52], and the cytokine/chemokine signature in the laboratory‐based cohort reassuringly reflected the clinical cohort. Lastly, in this exploratory study, we chose not to apply corrections for multiple analyte comparisons, as our primary aim was to explore analytes of potential clinical interest and refine hypotheses for future investigations. This approach can lead to false positives, and thus the raw *p*‐values should be interpreted within this exploratory context, emphasizing the need for further targeted studies to validate our findings.

Our findings highlight the potential applications of cytokine/chemokine and NfL testing in patients with LGI1‐AE, suggesting the importance of IL‐6, IL‐8/CXCL8, and IL‐17A in disease mechanisms and demonstrating their potential diagnostic, prognostic, and therapeutic value. Future studies evaluating the prognostic value of serum and CSF biomarkers in relation to LGI1‐AE clinical outcomes should utilize a prospective study design with age and sex matching, ensure sample collection at the time of diagnosis, limit pre‐exposure to immunotherapy, and consider consecutive longitudinal sample testing throughout the disease course and treatment period.

## Author Contributions

A.A., G.M., and A.Z. contributed to the conception and design of the study. All authors contributed to the acquisition and analysis of the data. A.A., G.M., and A.Z. contributed to drafting a significant portion of the manuscript or figures.

## Conflicts of Interest

A.A., G.M., B.Y., V.K.P., J.W.B., E.P.F., G.S.D., C.L.H., A.S.L., J.R.M., Y.H., and M.T. report no relevant disclosures related to this manuscript. D.D. has consulted for UCB, Immunovant, Argenx, Arialys, and Astellas pharmaceuticals. All compensation for consulting activities is paid directly to Mayo Clinic. He is a named inventor on a filed patent that relates to KLHL11 as a marker of autoimmunity and germ cell tumor. He has patents pending for LUZP4‐IgG, cavin‐4‐IgG, and SKOR2‐IgG as markers of neurological autoimmunity. He has received funding from the DOD (CA210208 & PR220430), the David J. Tomassoni ALS Research Grant Program, and UCB. E.P.F. has served on advisory boards for Alexion, Genentech, Horizon Therapeutics, and UCB. He has received research support from UCB. He received royalties from UpToDate. Dr. Flanagan is a site principal investigator in a randomized clinical trial of Rozanolixizumab for relapsing myelin oligodendrocyte glycoprotein antibody‐associated disease run by UCB. Dr. Flanagan is a site principal investigator and a member of the steering committee for a clinical trial of satralizumab for relapsing myelin oligodendrocyte glycoprotein antibody‐associated disease run by Roche/Genentech. Dr. Flanagan is a Co‐Investigator on a study of ravulizumab for neuromyelitis optica spectrum disorder run by Alexion. Dr. Flanagan has given educational talks on neuromyelitis optica spectrum disorder funded by Alexion. Dr. Flanagan has received funding from the NIH (R01NS113828). Dr. Flanagan has received honoraria for editing and writing articles for The Continuum Lifelong Learning in Neurology Journal, which is a publication of the American Academy of Neurology. Dr. Flanagan is a member of the medical advisory board of the MOG project. Dr. Flanagan is an editorial board member of Neurology, Neuroimmunology and Neuroinflammation, The Journal of the Neurological Sciences, and Neuroimmunology Reports. A patent has been submitted on DACH1‐IgG as a biomarker of paraneoplastic autoimmunity. S.R.I. has received honoraria/research support from UCB, Immunovant, MedImmun, Roche, Janssen, Cerebral therapeutics, ADC therapeutics, BioHaven therapeutics, CSL Behring, and ONO Pharma and receives licensed royalties on patent application WO/2010/046716 entitled ‘Neurological Autoimmune Disorders’, and has filed two other patents entitled “Diagnostic method and therapy” (WO2019211633 and US app 17/051,930; PCT application WO202189788A1) and “Biomarkers” (WO202189788A1, US App 18/279,624; PCT/GB2022/050614). A.M. has patents issued for GFAP and MAP1B‐IgGs and patents pending for Septins‐5 and Septins‐7, and KLCHL11‐IgGs; and has consulted for Roche pharmaceuticals, without personal compensation. I.V. is a full‐time employee of F. Hoffmann‐La Roche Ltd. S.J.P. has received personal compensation for serving on scientific advisory boards or data safety monitoring boards for F. Hoffman‐LaRoche AG, Genentech, Arialys, and UCB. His institution has received compensation for serving as a consultant for Astellas, Alexion/AstraZeneca, and Viela Bio/MedImmune/Amgen. All compensation is paid to Mayo Clinic. He has received research support from Alexion/AstraZeneca, Viela Bio/MedImmune/Amgen, Roche/Genentech, and Adimmune. He has a patent, Patent# 8,889,102 (Application#12–678,350, Neuromyelitis Optica Autoantibodies as a Marker for Neoplasia)—issued; a patent, Patent# 9,891,219B2 (Application#12–573,942, Methods for Treating Neuromyelitis Optica (NMO) by Administration of Eculizumab to an individual that is Aquaporin‐4 (AQP4)‐IgG Autoantibody positive)‐issued and from which he has received royalties; and a patent for GFAP‐IgG, Septin‐5‐IgG, MAP1B‐IgG, Kelch‐like protein 11, and PDE10A pending. He is working as a consultant in the Mayo Clinic Neuroimmunology laboratory clinical service. The Mayo Clinic Neuroimmunology Laboratory commercially offers MOG‐IgG testing, but revenue accrued does not contribute to salary, research support, or personal income. A.Z. has patents submitted for DACH1‐IgG, PDE10A‐IgG, CMAKV‐IgG, Tenascin‐R‐IgG as biomarkers of neurological autoimmunity. She has received research funding from Roche/Genentech and the Center for MS and Autoimmune Neurology at Mayo Clinic relevant to this manuscript. She has consulted without personal compensation for Alexion Pharmaceuticals.

## Supporting information


**Table S1:** Comparison of analyte levels between the clinical and laboratory‐based LGI1‐AE cohorts.

**Table S2:** Comparison of analyte levels between prospectively and retrospectively collected samples in the clinical LGI1‐AE cohort.

**Table S3:** Logistic Regression Model Estimates and Predictive Performance of Biomarkers.

## Data Availability

The data that support the findings of this study are available on request from the corresponding author. The data are not publicly available due to privacy or ethical restrictions.
