# Supplementary material for: Cytokine, Chemokine, and Neurofilament Light Chain Signatures in LGI1 Autoimmune Encephalitis
Source: Ann Clin Transl Neurol. 2025 Aug 8;12(11):2258–70. doi: 10.1002/acn3.70158 (PMC12623847; doi:10.1002/acn3.70158)
Supplement: Supplementary file 1 — Table S1: Comparison of analyte levels between the clinical and laboratory‐based LGI1‐AE cohorts. [file ACN3-12-2258-s003.docx]

| **Table S1. Comparison of analyte levels between the clinical and laboratory-based LGI1-AE cohorts** | | | | | | |
| --- | --- | --- | --- | --- | --- | --- |
|  | **Clinical (N=44)** | | | **Laboratory-based (N=58)** | | |
| **Male sex, n (%)** | 29 (66) | | | 29 (50) | | |
| **Median age at collection, years (range)** | 69.5 (8-85) | | | 68 (16-86) | | |
| **Analyte** | **Median, pg/mL (IQR)** | **Elevated (%)** | **P-value**^1^ | **Median, pg/mL (IQR)** | **Elevated (%)** | **P-value**^1^ |
| CSF IL-1-beta | 0.3 (0.2, 0.3) | 2/41 (5) | n/a^2^ | 0.2 (0.2, 0.4) | 2/58 (3) | n/a^2^ |
| CSF IL-2 | 0.2 (0.1, 0.3) | 2/44 (5) | n/a^2^ | 0.2 (0.1, 0.3) | 2/58 (3) | n/a^2^ |
| CSF IL-4 | 0 (0, 0) | 1/44 (2) | n/a^2^ | 0 (0, 0.2) | 1/57 (2) | n/a^2^ |
| CSF IL-5 | 0.3 (0.1, 0.4) | 0 (0) | n/a^2^ | 0.4 (0.3, 0.5) | 2/58 (3) | n/a^2^ |
| CSF IL-6 | 2.6 (1.9, 3.7) | 2/43 (5) | n/a^2^ | 5.9 (2.6, 14.7) | 23/57 (40) | 0.007* |
| CSF IL-10 | 0.5 (0.2, 0.7) | 0/43 (0) | n/a^2^ | 0.7 (0.6, 1.1) | 9/57 (16) | 0.007* |
| CSF IL-12p70 | 0.3 (0.2, 0.4) | 0/40 (0) | n/a^2^ | 0.3 (0.2, 0.4) | 0/58 (0) | n/a^2^ |
| CSF IL-13 | 0 (0, 0) | 2/44 (5) | n/a^2^ | 0 (0, 0) | 1/57 (2) | n/a^2^ |
| CSF IL-17A | 1 (0.4, 1.4) | 5/41 (12) | n/a^2^ | 1.3 (0.9, 2) | 17/53 (32) | <0.001* |
| CSF BAFF | 115 (75, 173) | 1/44 (2) | n/a^2^ | 190 (116, 255) | 10/58 (17) | <0.001* |
| CSF IL-8/CXCL8 | 34 (20, 48) | 1/44 (2) | n/a^2^ | 68 (42, 115) | 17/58 (29) | <0.001* |
| CSF CXCL9 | 38 (23, 66) | 0/44 (0) | n/a^2^ | 78 (45, 156) | 5/58 (9) | n/a^2^ |
| CSF CXCL10 | 125 (22, 201) | 0/44 (0) | n/a^2^ | 182 (101, 324) | 9/58 (16) | 0.93 |
| CSF CXCL13 | 2.5 (0.9, 3.7) | 0/43 (0) | n/a^2^ | 4.6 (2.4, 10) | 5/58 (9) | n/a^2^ |
| CSF GM-CSF | 0.1 (0, 0.2) | 4/44 (9) | n/a^2^ | 0.3 (0, 0.5) | 5/56 (9) | n/a^2^ |
| CSF IFN-gamma | 0 (0, 0.1) | 4/29 (14) | n/a^2^ | 0.1 (0, 0.1) | 3/46 (7) | n/a^2^ |
| CSF TNF-alpha | 0.7 (0.3, 1) | 0/42 (0) | n/a^2^ | 0.8 (0.6, 1.3) | 4/56 (7) | n/a^2^ |
| Serum IL-1-beta | 0.1 (0.1, 0.2) | 9/20 (45) | 0.002*^3^ | 0.1 (0.1, 0.3) | 14/30 (47) | <0.001*^3^ |
| Serum IL-2 | 0.1 (0, 0.2) | 1/20 (5) | n/a^2^ | 0.1 (0.1, 0.2) | 1/30 (3) | n/a^2^ |
| Serum IL-4 | 0 (0, 0) | 1/20 (5) | n/a^2^ | 0 (0, 0) | 1/30 (3) | n/a^2^ |
| Serum IL-5 | 0.4 (0.2, 0.6) | 4/20 (20) | 0.05 | 0.4 (0.2, 0.4) | 5/30 (17) | 0.23 |
| Serum IL-6 | 4 (2.8, 9) | 16/20 (80) | <0.001* | 6.2 (3.5, 11.9) | 26/29 (90) | <0.001* |
| Serum IL-10 | 1.4 (1, 2) | 0/20 (0) | n/a^2^ | 1.2 (1, 2) | 3/29 (10) | n/a^2^ |
| Serum IL-12p70 | 0.6 (0.4, 0.9) | 8/19 (42) | 0.03*^3^ | 0.5 (0.3, 1.1) | 11/29 (38) | 0.02*^3^ |
| Serum IL-13 | 0 (0, 2.1) | 1/20 (5) | n/a^2^ | 0.1 (0, 1.2) | 1/29 (3) | n/a^2^ |
| Serum IL-17A | 0.9 (0.4, 1.7) | 9/19 (47) | 0.006* | 1.3 (0.9, 1.9) | 18/28 (64) | <0.001* |
| Serum BAFF | 514 (400, 618) | 4/20 (20) | 0.98 | 441 (313, 523) | 3/30 (10) | n/a^2^ |
| Serum IL-8/CXCL8 | 16 (10, 21) | 6/20 (30) | 0.22 | 17 (14, 42) | 13/30 (43) | 0.01* |
| Serum CXCL9 | 955 (349, 1220) | 7/20 (35) | 0.06 | 587 (366, 923) | 7/30 (23) | 0.05 |
| Serum CXCL10 | 148 (103, 178) | 0/20 (0) | n/a^2^ | 62 (39, 108) | 2/30 (7) | n/a^2^ |
| Serum CXCL13 | 36 (20, 52) | 0/20 (0) | n/a^2^ | 20 (12, 28) | 0/30 (0) | n/a^2^ |
| Serum GM-CSF | 0.6 (0.2, 0.9) | 4/19 (21) | 0.04*^3^ | 0.4 (0.2, 1.1) | 8/30 (27) | <0.001*^3^ |
| Serum IFN-gamma | 0.7 (0.4, 1.5) | 2/20 (10) | n/a^2^ | 0.5 (0.3, 1.8) | 5/30 (17) | 0.60 |
| Serum TNF-alpha | 9.9 (7.2, 12) | 3/20 (15) | 0.65 | 6.6 (5.3, 7.2) | 1/30 (3) | n/a^2^ |
| ^1^Wilcoxon rank sum test (compared to mixed non-inflammatory controls)  ^2^Analyte elevated in <15% of patients within the subgroup  ^3^Median value below the lower limit of quantitation of the assay  *Statistically significant (p<0.05) | | | | | | |
