# Supplementary material for: Cytokine, Chemokine, and Neurofilament Light Chain Signatures in LGI1 Autoimmune Encephalitis
Source: Ann Clin Transl Neurol. 2025 Aug 8;12(11):2258–70. doi: 10.1002/acn3.70158 (PMC12623847; doi:10.1002/acn3.70158)
Supplement: Supplementary file 2 — Table S2: Comparison of analyte levels between prospectively and retrospectively collected samples in the clinical LGI1‐AE cohort. [file ACN3-12-2258-s002.docx]

| **Table S2. Comparison of analyte levels between prospectively and retrospectively collected samples in the clinical LGI1-AE cohort** | | | |
| --- | --- | --- | --- |
|  | **Prospective (N=14)** | **Retrospective (N=30)** |  |
| **Analyte** | **Median, pg/mL (IQR)** | **Median, pg/mL (IQR)** | **P-value**^1^ |
| CSF IL-1-beta | 0.3 (0.2, 0.5) | 0.2 (0.2, 0.3) | 0.09 |
| CSF IL-2 | 0.3 (0.2, 0.3) | 0.2 (0.1, 0.3) | 0.02*^2^ |
| CSF IL-4 | 0 (0, 0) | 0 (0, 0) | 0.02*^2^ |
| CSF IL-5 | 0.3 (0.2, 0.4) | 0.3 (0.1, 0.4) | 0.45 |
| CSF IL-6 | 3.3 (2.5, 3.8) | 2.5 (1.4, 3.6) | 0.17 |
| CSF IL-10 | 0.6 (0.4, 0.8) | 0.3 (0.1, 0.6) | 0.03* |
| CSF IL-12p70 | 0.3 (0.2, 0.4) | 0.3 (0.2, 0.4) | 0.93 |
| CSF IL-13 | 0 (0, 0) | 0 (0, 0) | 0.29 |
| CSF IL-17A | 0.7 (0.5, 1.2) | 1 (0.4, 1.7) | 0.26 |
| CSF BAFF | 136 (100, 164) | 104 (67, 189) | 0.28 |
| CSF IL-8/CXCL8 | 36 (29, 46) | 30 (18, 48) | 0.31 |
| CSF CXCL9 | 55 (32, 61) | 29 (19, 70) | 0.30 |
| CSF CXCL10 | 165 (127, 211) | 69 (0.9, 164) | 0.01* |
| CSF CXCL13 | 2.9 (1.1, 3.8) | 1.8 (0.6, 3.5) | 0.46 |
| CSF GM-CSF | 0.1 (0, 0.3) | 0.1 (0, 0.2) | 0.92 |
| CSF IFN-gamma | 0 (0, 0.1) | 0 (0, 0.1) | 0.09 |
| CSF TNF-alpha | 0.8 (0.7, 1) | 0.5 (0.1, 0.9) | 0.02* |
| Serum IL-1-beta | 0.2 (0.1, 0.2) | 0.1 (0.1, 0.3) | 0.77 |
| Serum IL-2 | 0.2 (0.1, 0.2) | 0.1 (0, 0.2) | 0.71 |
| Serum IL-4 | 0 (0, 0.1) | 0 (0, 0) | 0.39 |
| Serum IL-5 | 0.3 (0.2, 0.5) | 0.5 (0.3, 0.6) | 0.32 |
| Serum IL-6 | 3.9 (2.4, 4.1) | 6.2 (2.9, 12.8) | 0.19 |
| Serum IL-10 | 2.2 (1.7, 2.6) | 1.1 (1, 1.6) | 0.02* |
| Serum IL-12p70 | 0.6 (0.5, 0.9) | 0.6 (0.3, 0.9) | 0.60 |
| Serum IL-13 | 0 (0, 5.3) | 0.1 (0, 1) | 0.72 |
| Serum IL-17A | 0.7 (0.3, 1.2) | 1 (0.5, 1.8) | 0.64 |
| Serum BAFF | 415 (352, 493) | 564 (448, 705) | 0.05 |
| Serum IL-8/CXCL8 | 16 (11, 19) | 15 (10, 25) | 0.87 |
| Serum CXCL9 | 973 (955, 1120) | 652 (262, 1296) | 0.56 |
| Serum CXCL10 | 157 (128, 202) | 142 (73, 173) | 0.46 |
| Serum CXCL13 | 64 (50, 83) | 23 (13, 37.6) | 0.003* |
| Serum GM-CSF | 0.5 (0.1, 0.9) | 0.6 (0.2, 0.9) | 0.73 |
| Serum IFN-gamma | 0.8 (0.4, 1.2) | 0.7 (0.4, 1.5) | 0.80 |
| Serum TNF-alpha | 12.9 (9.1, 16) | 8.6 (6.7, 11.7) | 0.10 |
| ^1^Wilcoxon rank sum test  ^2^Median values below the lower limit of quantitation of the assay  *Statistically significant (p<0.05) | | | |
