# Supplementary material for: Cytokine, Chemokine, and Neurofilament Light Chain Signatures in LGI1 Autoimmune Encephalitis
Source: Ann Clin Transl Neurol. 2025 Aug 8;12(11):2258–70. doi: 10.1002/acn3.70158 (PMC12623847; doi:10.1002/acn3.70158)
Supplement: Supplementary file 3 — Table S3: Logistic Regression Model Estimates and Predictive Performance of Biomarkers. [file ACN3-12-2258-s001.docx]

| **Table S3. Logistic Regression Model Estimates and Predictive Performance of Biomarkers** | | | | | | | |
| --- | --- | --- | --- | --- | --- | --- | --- |
| **Outcome Measure** | **Predictor^a^** | **Odds Ratio** | **p-value** | **AUC (Analyte + Covariate^a^)** | **AUC (Covariate-only^a^)** | **ΔAUC** | **p-value^b^** |
| **Any Relapse^c^** | IL-6 (log2) | 2.8^d^ | 0.02* | 0.71 | 0.50 | +0.21 | 0.009* |
| **Relapse after CSF collection^c^** | IL-6 (log2) | 2.6^d^ | 0.03* | 0.69 | 0.50 | +0.19 | 0.03* |
| **Chronic epilepsy^e^** | NfL (log2) | 1.9^d^ | 0.03* | 0.82 | 0.68 | +0.14 | 0.01* |
|  | Male Sex | 10.3 | 0.046* |  |  |  |  |
| The AUC values represent the predictive performance of models with and without the inclusion of analyte levels  *Statistically significant (p<0.05)  ^a^Covariates assessed: Sex, Age at collection, Immune therapy at collection, Prospective collection, Time from onset to treatment, Time from onset to collection  ^b^DeLong's test for two correlated ROC curves  ^c^New or worsening symptoms after ≥1 month of clinical stability  ^d^The estimates correspond to the predicted relative odds for a 2-fold increase in the original scale of the predictor, based on its log2-transformed value, while all other variables constant.  ^e^Presence of ongoing seizures in the last 6 months before last follow-up  AUC: Area under the curve; IL: interleukin; NfL: Neurofilament light chain | | | | | | | |
